# Gene Regulatory Networks and Signaling Pathways in Palatogenesis and Cleft Palate: A Comprehensive Review

**DOI:** 10.3390/cells12151954

**Published:** 2023-07-27

**Authors:** Hyung-Jin Won, Jin-Woo Kim, Hyung-Sun Won, Jeong-Oh Shin

**Affiliations:** 1 Department of Anatomy, School of Medicine, Kangwon National University, Chuncheon 24341, Republic of Korea; 2 BIT Medical Convergence Graduate Program, Department of Microbiology and Immunology, School of Medicine, Kangwon National University, Chuncheon 24341, Republic of Korea; 3 Graduate School of Clinical Dentistry, Ewha Womans University, Seoul 03760, Republic of Korea; 4 Department of Oral and Maxillofacial Surgery, School of Medicine, Ewha Womans University, Seoul 03760, Republic of Korea; 5 Department of Anatomy, Wonkwang University School of Medicine, Iksan 54538, Republic of Korea; 6 Jesaeng-Euise Clinical Anatomy Center, Wonkwang University School of Medicine, Iksan 54538, Republic of Korea; 7 Department of Anatomy, College of Medicine, Soonchunhyang University, Cheonan 33151, Republic of Korea; 8 BK21 FOUR Project, College of Medicine, Soonchunhyang University, Cheonan 33151, Republic of Korea

**Keywords:** palate development, congenital disorder, cleft palate/lip, genetic network

## Abstract

Palatogenesis is a complex and intricate process involving the formation of the palate through various morphogenetic events highly dependent on the surrounding context. These events comprise outgrowth of palatal shelves from embryonic maxillary prominences, their elevation from a vertical to a horizontal position above the tongue, and their subsequent adhesion and fusion at the midline to separate oral and nasal cavities. Disruptions in any of these processes can result in cleft palate, a common congenital abnormality that significantly affects patient’s quality of life, despite surgical intervention. Although many genes involved in palatogenesis have been identified through studies on genetically modified mice and human genetics, the precise roles of these genes and their products in signaling networks that regulate palatogenesis remain elusive. Recent investigations have revealed that palatal shelf growth, patterning, adhesion, and fusion are intricately regulated by numerous transcription factors and signaling pathways, including Sonic hedgehog (Shh), bone morphogenetic protein (Bmp), fibroblast growth factor (Fgf), transforming growth factor beta (Tgf-β), Wnt signaling, and others. These studies have also identified a significant number of genes that are essential for palate development. Integrated information from these studies offers novel insights into gene regulatory networks and dynamic cellular processes underlying palatal shelf elevation, contact, and fusion, deepening our understanding of palatogenesis, and facilitating the development of more efficacious treatments for cleft palate.

## 1. Introduction

Orofacial development is important for the growth and change of the face and mouth from embryo to adulthood. The lip and palate play a crucial role in vital functions such as breathing, speech, mastication, and respiration. Orofacial clefts, including cleft lip and palate, are among the most common congenital anomalies, affecting roughly 17 out of 10,000 live births in the United States [1]. These anomalies significantly impact individuals and society, including lifetime costs estimated at USD 200,000, loss of productivity, increased use of mental health services, and increased morbidity and mortality [2,3]. Clinically, orofacial clefts can be classified as syndromic or non-syndromic based on associated anomalies and/or medical conditions beyond the cleft itself [4,5,6]. A mouse model is commonly used in research on palate development and defect due to its genetic advantages and the availability of multiple genetic modification methods and resources. Recent advances in molecular biology and mouse genetics have improved our understanding of the fundamental mechanisms involved in orofacial development [7,8,9]. A thorough understanding of the cleft palate is critical for advancing the field of developmental biology and enabling optimal prevention, treatment, and prognosis of these conditions. This review discusses progress and pathological mechanisms, focusing on genetic pathways and mechanisms of palatogenesis at cellular and molecular levels.

## 2. Morphologenetic and Molecular Mechanisms in Mammalian Palatogenesis

### 2.1. Anatomical Overview of Palatogenesis

The palate is divided into two main parts: the hard palate (the anterior bony part) and the soft palate (the posterior muscular part). It separates oral and nasal cavities. During embryogenesis, palatal shelves gradually elevate and come together at the midline, forming the hard palate. The soft palate is formed by soft tissues located posterior to the hard palate, separating the nasal and oral cavities during speech and swallowing. Fusion of palatal shelves begins at their posterior edge and progresses anteriorly [10].

Facial development in humans starts around the fourth week of gestation. The five facial primordia consist of the frontonasal prominence, two mandibular prominences, and two maxillary prominences. Facial development takes place between the fifth and twelfth weeks during embryogenesis. The frontonasal prominence is divided into lateral and medial nasal processes by formation of nasal pits, which subsequently fuse to form the nostril [5,10]. Palatal shelves comprise cranial neural crest cells derived from mesenchyme and oral epithelium. Face development requires coordination of a series of formal events, including cell growth, migration, differentiation, and death [11].

The upper lip, philtrum, and primary palate are formed by the union of medial nasal processes and maxillary processes [12]. Their disruptions during the fusion process can lead to cleft lip and/or palate formation. Prospective secondary palates are presented from the oral side of maxillary processes. The secondary palate develops as paired protrusions that grow vertically with the growing tongue and reorient to a horizontal position across the dorsal portion of the tongue in a process known as palatal shelf elevation. Palatal shelves expand toward the midline, resulting in contact and fusion at this location. The perfect fusion of palatal shelves on each side involves formation of a midline epithelial seam and its disappearance to fill mesenchymal cells. Next, the secondary palate undergoes fusion at its anterior aspect with the primary palate and at its anterodorsal area with the nasal septum, which derives from medial nasal processes at the same period. Finally, the intact roof of the oral cavity is developed, and the oral cavity and nasal cavity are separated. Failure of palatal shelf elevation, contact, and adhesion causes secondary cleft palate. In humans, the development of palate begins at the sixth week of gestation. It is fully accomplished by the twelfth week [10]. In mice, palatal growth is detected at embryonic day (E) 11.5, with palatal fusion being completed by E17 [11,12,13]. Palatogenesis depends on the precise temporal–spatial control of genetic components such as growth factors and signaling molecules for proper development. Several factors including maternal smoking or substance abuse and exposure to environmental toxins can affect palate development. Orofacial clefts result from disruptions of normal biomolecular processes of craniofacial development.

### 2.2. Classification of Cleft Lip and Palate in Human

**Figure 1:**
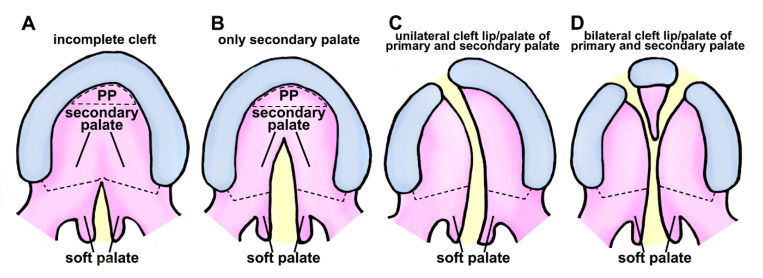
**Schematic representation of cleft palate/lip classification in human.** (**A**) Class I is characterized by an inadequate separation in the palate region, affecting only the soft tissue. (**B**) Class II clefts impact the secondary palate, consisting of hard and soft palate components. (**C**) Unilateral separation of primary and secondary palate components characterizes class III. (**D**) Class IV clefts are characterized by a complete bilateral separation affecting primary and secondary palates. Black dotted semicircular-shape, primary palate (PP); black dotted line, the boundary of the hard palate and soft palate; yellow area represents clefting region.

In human, cleft lip and palate are common congenital disabilities that can affect the structure of the face. There are several classifications for describing clefts of the palate and lip. Cleft lip is a congenital condition that occurs when tissues of the upper lip do not fuse together properly during human embryonic development. This can result in a gap or opening in the lip, which can range in size and location. In addition, cleft lip can occur on one side of the lip (unilateral cleft lip) or on both sides of the lip (bilateral cleft lip) (Figure 1).

Unilateral cleft lip is further classified based on the extent of the cleft and location of the cleft within the lip. Unilateral cleft lip of human has three subtypes:

Incomplete cleft lip: This type of cleft lip is characterized by a gap or opening in the lip that is smaller than a complete cleft lip. The location and size of the cleft can vary.

Complete cleft lip: This type of cleft lip involves the entire width of the upper lip, extending from the base of the nose (boundary between the lip and the surrounding skin).

Median cleft lip: This type of cleft lip is a rare type of unilateral cleft lip that occurs in the center of the upper lip, dividing the lip into two separate halves.

Bilateral cleft lip is a type of cleft lip that involves both sides of the upper lip. This type of cleft lip is less common than unilateral cleft lip. It can be more challenging to treat due to the extent of deformity.

Cleft palate is an aberration that arises when the roof of the mouth fails to fuse correctly during human embryonic development. This can result in an opening in the roof of the mouth, which can range in size and location. Cleft palate can occur as a complete cleft (involving both the hard palate and the soft palate) or an incomplete cleft (involving either the hard palate or the soft palate). Unilateral cleft palate has three subtypes (Figure 1):

Complete cleft palate—involves both hard and soft palates.

Incomplete cleft palate—involves either the hard palate or the soft palate.

Submucous cleft palate—involves a small opening in the soft palate, with mucous membrane remaining intact.

Submucous cleft palate is a type of cleft palate that results from a small opening in the soft palate with an intact mucous membrane. This makes it more challenging to diagnose than other cleft palate forms. Consequently, diagnosis might be delayed until speech or hearing issues arise. Cleft lip and palate often occur together.

However, some individuals might have an asymmetric cleft affecting one side of the lip/palate or both sides in opposite configurations, which could impact classification and management [14,15]. Evidence involving mouse models specifically for asymmetry in orofacial clefts is limited; however, the formation of orofacial structures can be regulated not only by genetic factors but also by epigenetics and environmental factors. Despite substantial advancements in understanding the genetic etiology of orofacial clefts and accelerated identification of candidate causal mutations through technological and bioinformatic progress, clinical care and prevention strategies remain largely unaffected. This is primarily due to the limited comprehension of the cellular, molecular, and developmental processes underlying cleft pathogenesis [2,16,17]. Therefore, elucidating the causes through various mouse models is crucial for the development of treatments.

### 2.3. Morphological and Molecular Control of Palatal Shelf Growth and Patterning

Lip closure and palatal fusion during human gestation occur at the sixth and twelfth weeks [12,17], respectively, necessitating the use of animal models to study normal and abnormal craniofacial development [2,11,12,13,18,19]. The mouse serves as the primary model organism for investigating orofacial cleft pathogenesis, due to its genetic homology with humans, similar embryonic facial and palate development processes, and the availability of mouse strains with spontaneous or engineered mutations causing cleft lip and/or cleft palate phenotypes [12,19,20].

**Figure 2:**
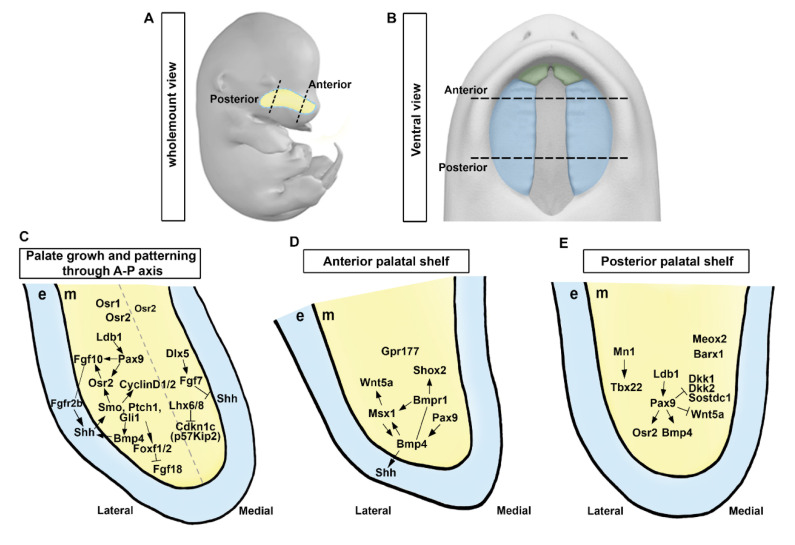
**Molecular regulation and signaling circuits in palatal shelf growth and patterning.** (**A**,**B**) A schematic diagram showing the wholemount view of the embryo and the ventral view of the developing palate at E13.5. (**C**) Signaling pathways governing palatal shelf growth and patterning along the anterior-posterior axis. (**D**,**E**) Regulation of growth in anterior and posterior regions of the palatal shelf involves specific molecular pathways, respectively. e, epithelium; m, mesenchyme. Black dotted lines, the anterior and posterior regions in the developing palate at E13.5; Light blue dotted line, developing palate; Gray dotted line, the hypothetical margin of the lateral and medial side in the palatal shelf. Arrows represent inductive relationships. Solid lines represent direct physical interaction. Blunt arrows indicate inhibition.

As we begin this chapter, our focus is on elucidating the mechanisms of palatogenesis through an in-depth exploration of palatal development in mice. Palatal shelves are composed mostly of neural crest-derived mesenchyme [21]. They are bordered by a thin layer of oral epithelium with a unique anterior–posterior (A–P) axis (Figure 2A,B). At E11.5, the palate is in the early stage of development. At this stage of development, the formation of palatal shelves and the frontonasal process have not yet begun, although neural crest cells that will form palatal shelves have already initiated migration to their proper location. The first signs of the maxillary process can also be seen at this stage. In E12.5, the frontonasal process and palatal shelves have not yet started to lengthen. Around E13.5, palatal shelves, which are two vertically oriented plates of tissue located in the roof of the mouth, begin to elevate and to move towards each other. The frontonasal process located at the front of the head contributes to the formation of the face and elongates to aid in outgrowth [11,13,22]. The elevating palatal shelves eventually come into contact and fuse in the midline. This process typically occurs between E13.5 and E15.5 [11]. Recent studies have shown that palatal shelf growth is regulated by reciprocal epithelial–mesenchymal interactions involving molecular mechanisms along the A–P axis [7,8,9,23,24].

Previous studies have identified essential components of signaling pathways for palatal shelf outgrowth. Several signaling molecules, including sonic hedgehog (Shh), wingless and Int-1 (Wnt), fibroblast growth factor (Fgf), and bone morphogenetic protein (BMP), can regulate palatal shelf outgrowth, with Shh playing a key role in palatal shelf outgrowth [2,8,9]. Inactivation of Shh in the epithelium or mesenchyme-specific inactivation of *Smoothened* (*Smo*) can impair palatal cell proliferation and outgrowth, indicating that Shh signaling is vital for mitogenic response of palatal cells [25]. Evidence for the role of SHH signaling in facial growth has been demonstrated by manipulating Hhat, which encodes an acyltransferase involved in modifying Hh proteins, and Ptch1 [26]. Mice with concurrent *Hhat* and *Ptch1* mutations displayed SHH gradient disruptions during frontonasal process development, resulting in medial and lateral nasal process hypoplasia and ultimately causing cleft lip and remaining midline epithelial seam [26]. Primary cilia are tiny hair-like projections that extend from the surface of many tissues in the body [27]. They play an important role in transmitting Shh signaling, as evidenced by reduced expression of *forkhead box F1* (*Foxf1*) in the palatal mesenchyme [28,29,30], suggesting that primary cilia might function as downstream effectors of Shh signaling. *Fgf10^−/−^* can lead to cleft palate with impaired palatal shelf outgrowth [31]. Although *Fgf10* expression is in the mesenchyme, its receptor *Fgfr2b* is detected in the overlying epithelium. Fgfr2 is required in the epithelium since an epithelial-specific deletion of *Fgfr2* also leads to cleft palate [32]. Odd-skipped related 2 (*Osr2*) is decreased in the palatal mesenchyme when Shh signaling is deleted [33]. *Osr2* expression in palatal mesenchymal cells depends on the function of *Pax9* [34,35]. Embryos with *Osr2^−/−^*; *Pax9^−/−^* exhibit cleft palate and reduction of *Fgf10* expression in the palatal mesenchyme [34,35]. Indeed, Shh is intensively reduced in the epithelium of *Fgf10^−/−^* and *Fgfr2b^−/−^* embryo, indicating that decreased palatal mesenchymal proliferation observed in these mutants might be due to decreased Shh expression in the epithelium [31]. Shh signaling acts in concert with FGF signaling by a positive feedback loop to control palatal epithelial and mesenchymal proliferation [31,33] (Figure 2C). Together, these two signaling pathways and transcription factors ensure proper formation of the palate and establishment of oral and nasal cavities by activating a mesenchymal signal (Figure 2C–E).

*Fgf10* plays a role in maintaining Shh expression in the palatal epithelium [31], whereas *Fgf7*, a closely related member of the FGF family, has the opposite effect by suppressing *Shh* expression in the palatal epithelium [36]. *Fgf7* expression in the medial palatal mesenchyme is regulated by *Dlx5*, which restricts *Shh* expression to the lateral palatal epithelium [36]. Emerging research on genetic and explant culture investigations has unveiled crucial molecular mechanisms underlying palatal shelf formation, which involve the intricate interplay among Shh, Foxf1/2, and Fgf18 within a complex regulatory network [24]. Ablation of both *Foxf1* and *Foxf2* in the neural crest of mouse embryos leads to defective development of palatal outgrowths and abnormal *Fgf18* expression in the palatal mesenchyme [24]. In addition, *Foxf2^−/−^* mouse embryos display restricted palatal shelf growth. Such defective growth is characterized by mislocalized, increased expression of *Fgf18* in specific regions of the palatal mesenchyme, and loss of *Shh* expression in complementary areas of the palatal epithelium [24]. Several transcription factors and unique FGF ligands, all of which are controlled by the Shh signaling pathway, coordinate many subnetworks that govern the growth and patterning of the palatal shelf [9] (Figure 2C).

In addition, LIM homeobox 6 (*Lhx6*) and LIM homeobox 8 (*Lhx8*) can regulate maxillary arch and palatal mesenchyme proliferation by suppressing expression of genes encoding FOX family transcription factors and *Cdkn1c* (also known as *p57Kip2*), a cell cycle inhibitor [37]. While the *Shh-Foxf1/2-Fgf18-Shh* molecular circuit has been recently found to be engaged in early development of the palate [24], it remains unknown whether *Lhx6/8* modulates Shh and FGF signaling network during formation of the palatal shelf (Figure 2C). Furthermore, transforming growth factor-β (TGF-β) signaling influences Shh signaling in the palatal mesenchyme by modulating lipid metabolism [38].

Shh and Bmp signaling pathways have been discovered to be able to interact with each other (Figure 2C,D). In the palatal mesenchyme, deletion of *Smo* leads to overexpression of *Bmp4* and downregulation of *Bmp2* [33]. Shh signaling can stimulate the expression of *Bmp2*, as confirmed by induction of *Bmp2* expression in palatal explant culture with Shh-containing beads [39]. Exogenous Bmp2 can also promote cell proliferation in the palatal mesenchyme [39]. Although complete inactivation of *Bmp4* was fatal during early stages of embryonic development, targeted ablation of *Bmp4* function solely in the maxillary mesenchyme and oral epithelium resulted in the development of cleft lip. At the same time, a no concomitant defect in the secondary palate was detected [40]. Palatal mesenchyme-specific overexpression of BMP antagonist Noggin in mice resulted in retarded palatal growth and cleft palate [41], further supporting the requirement for of BMP signaling for normal palatogenesis.

Previous studies identified that type I Bmp receptor *Bmpr1a* is a mediator of Bmp signaling in palate formation [40]. Deletion of *Bmpr1a* in the maxillary mesenchyme and oral epithelium of mice (in *Nestin-Cre; Bmpr1a^f/−^* mice) resulted in cleft lip and palate [40], while epithelial-specific loss of *Bmpr1a* (in *K14-Cre; Bmpr1a^f/−^*) did not cause cleft palate [42]. These findings suggest that Bmpr1a signaling in the palatal mesenchyme, rather than oral epithelium, is necessary for proper palatogenesis (Figure 2C). On the other hand, conditional deletion of *Bmpr1a* in the neural crest (in *Wnt1-Cre; Bmpr1a^f/−^* mouse embryos) and its derivatives can cause severe retardation in the anterior region of palatal shelves accompanied by various craniofacial defects [43]. Inactivation of *Bmpr1a* within the palatal mesenchyme was achieved using *Osr2-IresCre; Bmpr1a^f/f^* mice, which resulted in an anteriorly restricted cleft palate and decreased cell proliferation in the anterior palatal mesenchyme. In addition, loss of *Bmpr1a* in *Osr2-IresCre; Bmpr1a^f/f^* reduced cell proliferation and *Shh* expression in both primary and secondary palates, indicating that BMP–SHH interactions could regulate palate outgrowth [44]. Furthermore, loss of BMP antagonist Noggin caused cleft palate, with aberrant apoptosis in the palatal epithelium and reduced mesenchymal cell proliferation [45]. This demonstrates that strict regulation of BMP signaling is required for normal palate development (Figure 2C).

WNT signaling is vital in *Pax9*-mediated secondary palate development [9,23,46,47,48]. In *Pax9^−/−^* mice, posterior palate levels of *Axin2* and activated *β-catenin*—direct targets of canonical WNT signaling—were diminished, coinciding with increased WNT antagonist *Dkk2* expression [9,46]. Pharmacological inhibition of *DKK* activity using small-molecule agonists IIIc3a [9,46] or WAY-262611 [48] partially rescued palate morphology and fusion, leaving a minor cleft. Despite decreased *Sostdc1* and *Bmp4* expression in *Pax9^−/−^* mice, genetic inactivation of Sostdc1 sufficiently restored canonical WNT signaling in the palatal mesenchyme and rescued cleft palate [9,46].

In other developmental contexts, EDA/EDAR signaling is downstream of WNT signaling, and *Eda* expression is reduced in *Pax9^−/−^* mice [47]. Although EDA/EDAR signaling is not essential for palate formation [49], in utero stimulation with an EDAR agonist rescued cleft palate in *Pax9^−/−^* mice [47]. Treated mice exhibited disorganized rugae and unaltered *Bmp4*, *Msx1*, *Fgf10*, and *Osr2* expression, while WNT pathway component analysis was not performed. These studies imply that *Pax9* influences WNT signaling through modulation of WNT antagonists in the palatal mesenchyme, although further investigation is needed to understand the transcriptional regulation of WNT target genes.

### 2.4. Regionalization of Anterior and Posterior Palatal Outgrowth

Developing palatal shelves exhibit distinct molecular and morphological differences along the A–P axis, with specific pathways functioning in anterior and posterior compartments [50,51,52] (Figure 2C–E). Developing palatal shelves exhibit differential gene expression along the A–P axis, with various transcription factors including BarH-like homeobox 1 (*Barx1*), meningioma 1 (*Mn1*), Msh homeobox 1 (*Msx1*), mesenchyme homeobox 2 (*Meox2*), short stature homeobox 2 (*Shox2*), and T-box transcription factor 22 (*Tbx22*) being expressed in distinct regions [39,50,51,52,53,54]. For instance, *Msx1* and *Shox2* are expressed in the anterior palate, while *Meox2* and *Tbx22* are expressed in the posterior region, with the boundary aligned with the first-formed palatal rugae [39,50,51,52,53,54]. Transcription factors *Msx1* and *Shox2* are crucial for stimulating cellular proliferation in the anterior palatal mesenchyme where they are mainly expressed [39,54]. *Msx1* also plays a crucial role in maintaining Shh expression in the anterior palatal epithelium by regulating the expression of *Bmp4* in the anterior palatal mesenchyme [39]. *Barx1* and *Mn1* mRNAs are expressed mainly in the posterior palate. However, their expression domains also partially overlap with the anterior half of palatal shelves [52,55]. Mice with disrupted *Msx1* or *Mn1* gene expression exhibit a complete cleft palate while *Msx1^−^*^/*−*^ mice exhibit proliferation defects only in the anterior region and *Mn1^−^*^/*−*^ mice exhibit growth deficits confined to middle and posterior regions of the palatal shelves [39,55]. Conversely, *Shox2^−^*^/*−*^ animals show a cleft limited to the anterior palate, whereas the posterior palate shows normal fusion, indicating a particular function for *Shox2* in anterior palatal expansion [54] (Figure 2D). *Tbx22^−^*^/*−*^ mice displayed cleft palate, ranging from full cleft palate owing to shortened palatal shelves to the submucous cleft palate with normal palatal shelf elevation and fusion [56]. *Tbx22* mRNA expression is significantly reduced in *Mn1^−^*^/*−*^ mice and *Mn1* is capable of stimulating *Tbx22* expression in cell culture assays, indicating that *Tbx22* is downstream of *Mn1* in regulating posterior palatal outgrowth [55] (Figure 2E).

Proper regulation of *Msx1* and *Shox2* expression in the anterior palatal mesenchyme depends on Bmp signaling, as demonstrated by marked decreases of expression levels of both genes in the anterior palate of mice with *Wnt1-Cre; Bmpr1a^f/−^* genotype [43]. Remarkably, in palatal explant cultures, *Msx1* expression was induced only in the anterior palatal mesenchyme by adding Bmp4-soaked beads [50], whereas *Shox2* mRNA expression in palatal mesenchyme explants could not be induced by exogenous Bmp4 [54]. However, anterior palatal epithelium induced ectopic *Shox2* mRNA expression in the posterior palatal mesenchyme [54]. These results reveal intrinsic distinctions between the epithelium and mesenchyme along the A–P axis (Figure 2D).

Canonical Wnt signaling in the developing palatal mesenchyme is restricted to the anterior area, as identified by the *BATGAL* transgenic reporter and canonical Wnt signaling is dependent on *Gpr177*-mediated Wnt secretion by neural crest-derived mesenchyme [57]. Furthermore, *Wnt5a* is highly expressed in the anterior palatal mesenchyme. It governs palatal mesenchyme migration and palatal shelf elongation [58]. Msx1 can promote transcriptional activation of *Wnt5a* in the anterior palatal mesenchyme via an enhancer that originates from a transposable element [59]. On the other hand, LIM domain-containing transcription factors require cofactor *Ldb1* for proper palatal shelf growth and A–P patterning. In particular, neural crest-specific inactivation of *Ldb1* can induce ectopic *Wnt5a* in the posterior palatal mesenchyme [60], indicating that certain LIM domain-containing transcription factors might play a significant role in the growth of palatal shelf and A–P patterning.

### 2.5. Patterning along the Mediolateral Axis

Patterning along the mediolateral axis of the palate involves establishment of distinct gene expression domains that are necessary for proper palatal development. This patterning along the mediolateral axis in palate development is significant because it builds an intricate network of signaling channels and gene domains required for optimal palatal growth and fusion (Figure 2C). During vertical outgrowth of developing palatal shelves, morphological and molecular heterogeneity is visible along the mediolateral axis; the oral side aligned with the lateral side after elevation of the palatal shelf. In addition, around E12, the lateral side of the developing palatal shelves commences production of palatal rugae, concurrent with restriction of *Shh* expression to the lateral palatal epithelium [30]. *Osr1* and *Osr2* are zinc-finger transcription factor encoding genes that demonstrate graded expression along the mediolateral axis of the developing palatal mesenchyme [34]. At E13.5, *Osr1* mRNA is limited to the lateral side. However, *Osr2* displays graded expression which is the strongest in the lateral mesenchyme. It progressively decreases toward the medial mesenchyme. Deletion of *Osr2* results in formation of cleft palate, which is accompanied by decreased cell proliferation on the medial side of the developing palatal shelves as well as the disruption of mediolateral patterning [61]. Partial functional redundancy of *Osr2* and *Osr1* is likely responsible for the necessity of *Osr2* in mediating cell proliferation, particularly on the medial side since cleft palate could be repaired in *Osr2*-deficient mice by replacing *Osr2* coding sequence with an *Osr1* cDNA [61]. In *Osr2^−/−^* mice, transcriptional profiling and expression analysis demonstrated an increase in osteogenesis-related genes, such as *Mef2c*, *Sox6*, *Sp7*, and various BMP ligands (*Bmp3*, *Bmp5*, and *Bmp7*) [62]. In addition, class-3 semaphorins (*Sema3a*, *Sema3d*, and *Sema3e*) were found to be ectopically expressed and identified as direct targets of *Osr2* (specifically *Sema3a* and *Sema3d*). These findings indicate that *Osr2* has a crucial role in controlling mesenchymal cell proliferation and fate by inhibiting premature osteogenesis and abnormal semaphorin expression. Nevertheless, more research is required to comprehend the role of semaphorins in palate development [62].

A pathway involving transcription factor *distal-less homeobox 5* (*Dlx5*) can regulate mediolateral patterning and palatal expansion. In the medial mesenchyme of the palatal shelf, *Dlx5* is co-expressed with *Fgf7. Fgf7* expression is markedly decreased in this region in *Dlx5*-deficient mutant palatal shelves [36]. In *Dlx5*-deficient mutant embryo, expansion of *Shh* expression into the medial palatal epithelium might be attributed to loss of *Fgf7*, as demonstrated by the ability of exogenous Fgf7 to inhibit *Shh* expression in palatal explant cultures [36]. While palate shelves are elevated and fused in *Dlx5*-deficient individuals, the oral part of the palate is dramatically enlarged, and a deformed soft palate is visible. Intriguingly, while *Msx1*-deficient mice showed decreased *Shh* expression in the anterior palate, compound mutant embryos lacking both *Dlx5* and *Msx1* displayed Shh expression in the medial palatal epithelium, which was able to compensate for cell proliferation defects associated with *Msx1* loss-of-function [36]. Collectively, these findings identify a novel pathway involving *Dlx5* and *Fgf7* in regulating mediolateral patterning and palate growth. However, given that mice lacking *Fgf7* did not show any apparent palatal defects [63], another signaling molecule might function downstream of *Dlx5*, possibly in conjunction with *Fgf7*, to modulate *Shh* expression in the palatal epithelium (Figure 2C).

### 2.6. Genetic Network Controlling Palatal Shelf Adhesion and Fusion

**Figure 3:**
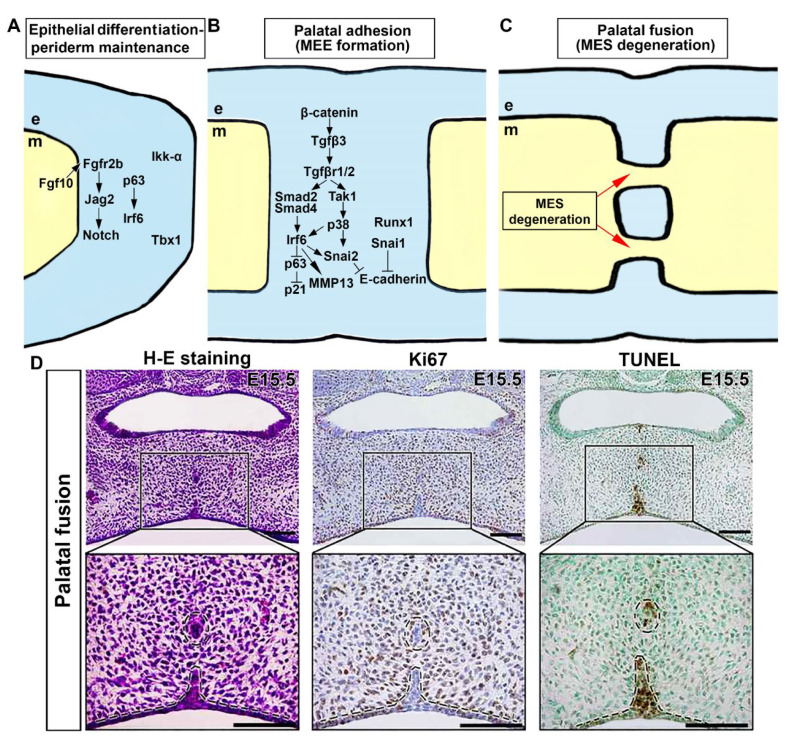
**Molecular and cellular processes underlying adhesion and fusion of the palate.** (**A**) Diverse intracellular pathways and transcription factors in the medial edge epithelium (MEE) and the periderm above instigate cell cycle exit, disturb epithelial adhesion, and degrade extracellular matrix (ECM). (**B**) Molecular control of palatal epithelial differentiation. (**C**) Morphological changes in midline epithelial seam (MES) during palatal fusion. (**D**) Histologic, cell proliferation, and cell death analysis of MES degeneration in the palatal fusion region at E15.5. Histological analysis indicates degeneration of MES during palatal fusion, with concurrent absence of cell proliferation and cell death in the remaining MES. Black, magnification of palatal fusion region [94]. Black dotted circle, remaining MES during palatal fusion. Black dotted line, the margin of palatal epithelium and mesenchyme. Arrows represent inductive relationships. Solid lines represent direct physical interaction. Blunt arrows indicate inhibition. e, epithelium; m, mesenchyme. Scale bar: 100 μm.

As the palatal shelf grows, maxillary and mandibular processes also grow. This enables the tongue to move downward and forward, which is necessary for elevating the palatal shelf. Upon their elevation, palatal shelves contact with each other at the midline and fuse [64]. A complex network of signaling pathways regulates the adhesion and fusion of the developing palatal shelves. Many important signaling pathways and genes are involved in epithelial differentiation of the palate. To establish mesenchymal continuity throughout the fused palate, it is necessary to eliminate the intervening epithelium between adjoining palatal shelves, which is referred to as midline epithelial seam (MES) (Figure 3). A cleft palate may result from disrupting spatial and temporal control of midline edge epithelium (MEE) differentiation, adhesion competence, and disappearance of MES.

Animals with malfunctioning *Jag2*, *Fgf10*, *Irf6*, and *Grhl3* genes have a cleft palate phenotype and aberrant adhesion or fusion of palatal shelves with the mandible and/or tongue [65,66,67,68,69]. Absence of *Jag2* Notch ligand can cause cleft palate in *Jag2z*^Δ*DSL/*Δ*DSL*^ mice primarily due to abnormal adhesion of palatal shelves to the tongue [70]. *Jag2* is expressed in the oral epithelium. It is responsible for maintaining periderm cells, which are believed to regulate fusion competence [66,71]. Moreover, palate–tongue fusion, although not severe, and decreased expression of *Jag2* in the palatal epithelium have been found in *Fgf10^−/−^* embryos, indicating that Fgf10 signaling can regulate palatal epithelial development upstream of Jag2-Notch signaling [65]. Mice lacking functional interferon regulatory factor 6 (*Irf6*) due to homozygous null mutations or an R84C point mutation show a hyperproliferative epidermis that does not differentiate, resulting in a range of developmental abnormalities, including cleft palate and inappropriate oral adhesions [67,69]. Irf6 can regulate periderm differentiation in collaboration with *Jag2*, as evidenced by the development of palate–tongue fusion, oral adhesions, and cleft palate in compound *Irf6^R84C/+^; Jag2^ΔDSL/+^* mice [72]. This phenotype resembles that seen in mice with homozygous *Irf6* or *Jag2* alleles, emphasizing the significance of these genes in palatal development. The expression of each gene was unaffected in the reciprocal individual mutant, indicating that Irf6 does not directly regulate *Jag2* expression [69].

It has been found that *p63* transcription factor-deficient mice exhibit a cleft palate and a thin, undifferentiated epidermis [73,74], with reduced Irf6 expression in the palatal epithelium [75]. In addition, heterozygous mutant mice, compound *p63^+/–^; Irf6^R84C/+^*, exhibit a failure in palatal shelf fusion associated with improper preservation of periderm cells. *p63* can exert a positive regulatory effect on the expression of *Jag2* and *Fgfr2* in various other cell types [76,77]. Although the relationship between p63, Jag2-Notch, and Fgf10-Fgfr2b signaling pathways in palatal epithelial differentiation is not yet fully understood, previous studies suggest that *p63* might positively regulate *Jag2* and *Fgfr2* expression in other cell types. In addition, a lack of *Ikk-α* or *Tbx1* in mouse embryos results in aberrant oral adhesions between the tongue and palatal shelves [78,79]. These findings suggest that palatal epithelial differentiation is regulated by a genetic network involving Irf6, Jag2, p63, Ikk-α, Tbx1, and Fgf10-Fgfr2b signaling pathways (Figure 3A).

Although periderm is essential to prevent abnormal oral epithelial adhesions, it must be removed from the medial edge of the palatal shelf to initiate fusion. Precise mechanisms responsible for controlling periderm removal have not yet been fully elucidated. The MES is the structure that separates palatal shelves prior to fusion. There are three main hypotheses explaining how the MES disappears. One hypothesis is that the MES disappears due to epithelial-to-mesenchymal transition (EMT), which could allow the intervening epithelium to be incorporated into the mesenchyme of the intact palate. For example, genetic lineage tracing using epithelial-restricted Cre-expressing transgenic lines paired with the ROSA26R (R26R) reporter line has been used to track the destiny of MES cells in vivo [80,81]. In one study, *lacZ* expression was specifically and irreversibly activated within the epithelium of *ShhGFPCre* or *K14-Cre* mice crossed with R26R reporter mice. Subsequent examination of β-galactosidase staining during and following MES removal allowed the fate of MEE cells to be followed to determine whether they contributed to the mesenchyme (i.e., if they underwent EMT) [80]. This approach did not detect *lacZ*-expressing mesenchymal cells, concluding that EMT was not a significant contributor to the regression of the MES [80]. However, a third group found mesenchymal β-galactosidase activity during and soon before regression of the MES in *K14-Cre; R26R* embryos [82]. The authors suggested that the disagreement might be due to variations in Cre expression levels and/or patterns in the palatal epithelium of several *K14-Cre* transgenic mice lines utilized.

Apoptosis has been shown to play a significant role in MES dissolution to obtain mesenchymal confluency. Cell proliferation is rarely observed at that location (Figure 3D). Several studies have shown that many MES cells are TUNEL positive and active caspase 3 positive during palatal fusion [80,83,84,85]. A new genetic research has studied the influence of the *Apaf1* gene, which encodes an essential component of caspase 3-mediated apoptosis, on palatal fusion and found that *Apaf1* deficiency does not impair palate fusion or MES dissolution [86]. This observation contrasts with a previous report indicating that palatal shelves could make contact but fail to fuse in *Apaf1*-deficient embryos [83]. However, that study did not perform a thorough evaluation of the secondary palate. Although apoptosis plays a substantial role in MES disintegration, further study is required to clarify the participation of other cellular processes, namely the fusion mechanism between the anterior secondary palate and primary and secondary palates. Despite this, the significance of TGF-β signaling in eliminating MES is clear since *Tgf-β3* is solely expressed in the MEE. The lack of *Tgf-β3* in embryonic mice allows palatal shelves to establish improper contact at the midline, resulting in persistence of the MES [87,88,89].

Contact between type I and type II receptor dimers can activate the Tgf-β signaling pathway, leading to phosphorylation of R-Smads and transcriptional regulation. *Smad2* knockdown in palatal explants can prevent the breakdown of the MES, while *Smad2* transgenic overexpression in the palatal epithelium partly repairs palate fusion in *Tgf-β3* deficient animals [90,91]. Nevertheless, epithelial-specific deletion of *Smad4* in *K14-Cre; Smad4^f/f^* mice did not affect palatal shelf fusion, suggesting the involvement of other pathways [91,92]. Tgf-β signaling may trigger the p38 MAPK pathway, which is increased in the palatal epithelium undergoing fusion. Tgf-β signaling triggers activation of *Tgf-β activated kinase 1* (*Tak1*), which operates separately from the Smad pathway, to initiate activation of the p38 MAPK pathway. Both pathways work redundantly to promote palatal fusion [11,92]. Inhibition of *p38 MAPK* in *K14-Cre; Smad4^f/f^* palatal explants prevents Tgf-β-dependent expression of the *p21* gene, reducing apoptosis and MES dissolution failure [92]. These findings suggest that *Smad-* and *p38 MAPK*-dependent mechanisms are functionally redundant during palate fusion. Notable, *Irf6*, a vital factor responsible for periderm differentiation, is activated not only in the periderm layer but also in the basal layer of MEE cells before fusion of palatal shelves [93]. Failure of palatal fusion and diminished MEE expression of Irf6 have been observed in *K14-Cre;Tgfβr2^f/f^* mutant embryos [93]. However, overexpression of Irf6 in basal epithelial cells has been found to restore palatal fusion in *K14-Cre;Tgfβr2^f/f^* embryos [93]. The *Irf6* expression functions during periderm differentiation, leading to the downregulation of *p63* and increased *p21* expression in MEE cells. This mechanism is believed to facilitate cell cycle exit and subsequent degeneration of the MEE [93,94] (Figure 3A). Tgf-β3 is crucial for downregulation of *Jag2* in the MEE. Blocking Notch signaling can partially restore fusion between *Tgf-β3*-deficient palatal shelves in explant culture [95]. Maintenance of oral periderm integrity depends on Jag2-Notch signaling [66]. Therefore, reduction of *Jag2* expression in the MEE is likely a key mechanism by which *Tgf-β3* disrupts periderm function and facilitates palatal shelf adhesion. A previous study revealed that beta-catenin (Ctnnb1) regulates MES dissolution by controlling *Tgf-β3* expression in the MEE. In epithelial-specific *beta-catenin* (*Ctnnb1*) disruption experiments, reduction of apoptotic MES cells and loss of *Tgf-β3* expression in the MEE were observed, resulting in cleft palate due to failed palatal shelf fusion [96]. However, given that *beta-catenin* (*Ctnnb1*) can function as either a component of adherent junctions or in the canonical Wnt signaling pathway [96], the precise mechanism underlying its involvement in this context remains to be elucidated through further investigation.

Several transcription factors play a crucial role in the initiation of palatal fusion (Figure 3A,B). The Snail family of transcription factors is essential in regulating palatal fusion, as evidenced by the failure of fusion in *Snai1^+/–^*; *Snai2^+/–^* compound mutants, which coincides with a reduction in MES apoptosis [97]. Expression of *Tgfβ-3* was not affected in these mutants [97], but exogenous Tgf-β3 in cultured primary MEE cells was found to induce the expression of *Snai1* via a pathway independent of Smad signaling. These results indicate that these transcription factors could regulate palatal fusion downstream of or in parallel with the Tgf-β3 pathway. Interestingly, *Runx1* is another transcription factor studied in the context of palate development (Figure 3A,B). Although it is expressed in the MEE throughout the AP axis during palate fusion, its disruption can lead to anterior-restricted failure of palatal shelf fusion and failed fusion with the primary palate. This anterior cleft is associated with a unique region of the MEE that exhibits less TUNEL staining and distinct behavior compared to the rest of the palatal shelf in unpaired palatal culture, suggesting that *Runx1* might play a role in this anterior MEE behavior [25]. In contrast, the *Meox2* transcription factor is required to maintain palatal integrity after successful fusion and dissolution of the MES. *Meox2^−/−^* embryos exhibit a post-fusion split of the posterior palate [86].

*Irf6* is required for *Snai2* expression in MEE cells. *Snai2* knockdown slows palatal fusion in explant cultures [98]. Activation of *Ephrin* reverse signaling enhances MEE expression of *Snai1* in palatal explant cultures and partly repairs palatal shelf fusion in the presence of *Tgf-β3* function-blocking antibodies [99]. These data imply that ephrin reverse signaling and Tgf-β3 signaling might cooperate to regulate palatal fusion. *Snai1* and *Snai2* are transcription factors that play a role in the epithelial–mesenchymal transition process. They act downstream of Tgf-β3 signaling. Snai family is known to downregulate the expression of *E-cadherin* [9]. This downregulation may contribute to loosening of the medial edge epithelium (MEE) and periderm cell adhesion, resulting in periderm desquamation. *Tgf-β3* and *Irf6* are crucial for activating the expression of *MMP13* in the palatal MEE, which may contribute to periderm desquamation by breaking down the basement membrane. In addition, carcinoembryonic antigen-related cell adhesion molecule 1 (*CEACAM1*) expression is upregulated in MEE periderm cells before palatal fusion and *Ceacam1^−/−^* mouse embryos display a delay in palatal fusion completion [100]. *Tgf-β3* expression in palatal MEE remains unaffected in *Ceacam1^−/−^* embryos. It is uncertain if *CEACAM1* functions downstream of *Tgf-β3* to regulate periderm desquamation and/or palatal shelf adhesion. The connection between molecular processes that lead to desquamation and apoptosis and other molecules involved in *Tgf-β3*-induced periderm cell apoptosis requires further investigation.

The genetic process of palatal development was described previously, and several studies additionally showed the role of epigenetic factors such as microRNAs that regulate genes in the palatal fusion process [101,102]. For example, miR-200b, highly expressed in epithelial cells [103], was found in the MES during palatal fusion and its expression decreased as fusion progressed. *Smad2*, essential for *Snai1* induction in Tgf-*β* signaling during palate development, was expressed in MEE and MES [101,102]. *Snai1*, crucial for palatal fusion via Tgf-*β* signaling, was present in mesenchyme and some MEE cells [102]. In addition, Ectopic miR-200b expression led to Zeb family suppression, *E-cadherin* upregulation, and alterations in cell migration and palatal fusion [101]. These findings indicate the critical role of miR-200b in cell migration and palatal fusion during palate development by regulating *Zeb1* and *Zeb2* as a noncoding RNA, while also suggesting a potential interaction with TGF-*β*-mediated *Smad2* and *Snai1* signaling pathways in the context of normal palate development.

While the MES ultimately undergoes degeneration, the epithelia situated on the nasal and oral facets of the palate differentiate into pseudo-stratified, ciliated columnar cells and stratified, squamous, keratinizing cells, respectively. Although epithelial differentiation is dictated by the subjacent mesenchyme [64], the molecular determinants governing oral and nasal epithelial cell fate remain elusive. Additionally, the palatal mesenchyme differentiates into osseous and muscular tissues, constituting the hard and soft palate, respectively; recently, reviews have explored the molecular mechanisms delineating these discrete cell fates [104,105].

## 3. Conclusions

Over the past quarter century, mouse genetics has considerably enhanced our knowledge of molecular processes that regulate palatal shelf development, patterning, and fusion, making it a helpful model for investigating genetic regulation of organogenesis in mammals. Remarkable heterogeneity and dynamism of molecular and cellular processes that occur across developing palatal shelves, along with the presence of signaling molecules and transcription factors, make it an exceptional model for studying genetic regulation of organogenesis in mammals. One of the challenges in this area of research pertains to acquiring in-depth knowledge about the direct mechanistic nature of relationships under investigation. Despite the well-established understanding of genetic pathways responsible for palatogenesis, biochemical mechanisms that underlie this process are still largely unexplored. To gain a comprehensive understanding of the molecular relationships involved in palate development, a combination of genetic, epigenetic, biochemical, and cell culture studies can be carried out using molecular and biochemical tools such as high-throughput gene expression profiling, mass spectrometry, and chromatin immunoprecipitation. As new technologies are being applied to investigate palatogenesis, our understanding of the fundamental principles controlling morphogenesis is rapidly advancing. The availability of new methodologies and tools will likely facilitate progress in understanding the molecular mechanisms governing palate development, ultimately providing crucial knowledge for understanding other organogenesis and birth defects, especially pathogenetic mechanisms underlying cleft palate syndrome.

## References

[1] Parker S.E., Mai C.T., Canfield M.A., Rickard R., Wang Y., Meyer R.E., Anderson P., Mason C.A., Collins J.S., Kirby R.S. (2010). Updated National Birth Prevalence estimates for selected birth defects in the United States, 2004–2006. Birth Defects Res. A Clin. Mol. Teratol..

[2] Leslie E.J., Marazita M.L. (2013). Genetics of Cleft Lip and Cleft Palate. Am. J. Med. Genet. C Semin. Med. Genet..

[3] Wehby G.L., Cassell C.H. (2010). The impact of orofacial clefts on quality of life and healthcare use and costs. Oral Dis..

[4] Carinci F., Pezzetti F., Scapoli L., Martinelli M., Avantaggiato A., Carinci P., Padula E., Baciliero U., Gombos F., Laino G. (2003). Recent developments in orofacial cleft genetics. J. Craniofac. Surg..

[5] Merritt L. (2005). Part 1. Understanding the embryology and genetics of cleft lip and palate. Adv. Neonatal Care.

[6] Mossey P.A., Little J., Munger R.G., Dixon M.J., Shaw W.C. (2009). Cleft lip and palate. Lancet.

[7] Lan Y., Jiang R. (2022). Mouse models in palate development and orofacial cleft research: Understanding the crucial role and regulation of epithelial integrity in facial and palate morphogenesis. Curr. Top. Dev. Biol..

[8] Hammond N.L., Dixon M.J. (2022). Revisiting the embryogenesis of lip and palate development. Oral Dis..

[9] Li C., Lan Y., Jiang R. (2017). Molecular and Cellular Mechanisms of Palate Development. J. Dent. Res..

[10] Patten B.M. (1964). Patten’s Foundation of Embryology.

[11] Bush J.O., Jiang R. (2012). Palatogenesis: Morphogenetic and molecular mechanisms of secondary palate development. Development.

[12] Jiang R., Bush J.O., Lidral A.C. (2006). Development of the upper lip: Morphogenetic and molecular mechanisms. Dev. Dyn..

[13] Gritli-Linde A. (2007). Molecular control of secondary palate development. Dev. Biol..

[14] Milling M.A., van Straelen P. (1996). Asymmetrical cleft palate. Br. J. Plast. Surg..

[15] Yuzuriha S., Oh A.K., Mulliken J.B. (2008). Asymmetrical bilateral cleft lip: Complete or incomplete and contralateral lesser defect (minor-form, microform, or mini-microform). Plast. Reconstr. Surg..

[16] Nasreddine G., El Hajj J., Ghassibe-Sabbagh M. (2021). Orofacial clefts embryology, classification, epidemiology, and genetics. Mutat. Res. Rev. Mutat. Res..

[17] Dixon M.J., Marazita M.L., Beaty T.H., Murray J.C. (2011). Cleft lip and palate: Understanding genetic and environmental influences. Nat. Rev. Genet..

[18] Chai Y., Maxson R.E. (2006). Recent advances in craniofacial morphogenesis. Dev. Dyn..

[19] Gritli-Linde A. (2008). The etiopathogenesis of cleft lip and cleft palate: Usefulness and caveats of mouse models. Curr. Top. Dev. Biol..

[20] Murray S.A. (2011). Mouse resources for craniofacial research. Genesis.

[21] Ito Y., Yeo J.Y., Chytil A., Han J., Bringas P., Nakajima A., Shuler C.F., Moses H.L., Chai Y. (2003). Conditional inactivation of Tgfbr2 in cranial neural crest causes cleft palate and calvaria defects. Development.

[22] Meng L., Bian Z., Torensma R., Von den Hoff J.W. (2009). Biological mechanisms in palatogenesis and cleft palate. J. Dent. Res..

[23] Jia S., Zhou J., D’Souza R.N. (2020). Pax9’s dual roles in modulating Wnt signaling during murine palatogenesis. Dev. Dyn..

[24] Xu J., Liu H., Lan Y., Aronow B.J., Kalinichenko V.V., Jiang R. (2016). A Shh-Foxf-Fgf18-Shh Molecular Circuit Regulating Palate Development. PLoS Genet..

[25] Charoenchaikorn K., Yokomizo T., Rice D.P., Honjo T., Matsuzaki K., Shintaku Y., Imai Y., Wakamatsu A., Takahashi S., Ito Y. (2009). Runx1 is involved in the fusion of the primary and the secondary palatal shelves. Dev. Biol..

[26] Kurosaka H., Iulianella A., Williams T., Trainor P.A. (2014). Disrupting hedgehog and WNT signaling interactions promotes cleft lip pathogenesis. J. Clin. Investig..

[27] Goetz S.C., Anderson K.V. (2010). The primary cilium: A signalling centre during vertebrate development. Nat. Rev. Genet..

[28] Everson J.L., Fink D.M., Yoon J.W., Leslie E.J., Kietzman H.W., Ansen-Wilson L.J., Chung H.M., Walterhouse D.O., Marazita M.L., Lipinski R.J. (2017). Sonic hedgehog regulation of Foxf2 promotes cranial neural crest mesenchyme proliferation and is disrupted in cleft lip morphogenesis. Development.

[29] Huangfu D., Liu A., Rakeman A.S., Murcia N.S., Niswander L., Anderson K.V. (2003). Hedgehog signalling in the mouse requires intraflagellar transport proteins. Nature.

[30] Shin J.O., Song J., Choi H.S., Lee J., Lee K., Ko H.W., Bok J. (2019). Activation of sonic hedgehog signaling by a Smoothened agonist restores congenital defects in mouse models of endocrine-cerebro-osteodysplasia syndrome. eBioMedicine.

[31] Rice R., Spencer-Dene B., Connor E.C., Gritli-Linde A., McMahon A.P., Dickson C., Thesleff I., Rice D.P. (2004). Disruption of Fgf10/Fgfr2b-coordinated epithelial-mesenchymal interactions causes cleft palate. J. Clin. Investig..

[32] Hosokawa R., Deng X., Takamori K., Xu X., Urata M., Bringas P., Chai Y. (2009). Epithelial-specific requirement of FGFR2 signaling during tooth and palate development. J. Exp. Zool. B Mol. Dev. Evol..

[33] Lan Y., Jiang R. (2009). Sonic hedgehog signaling regulates reciprocal epithelial-mesenchymal interactions controlling palatal outgrowth. Development.

[34] Lan Y., Ovitt C.E., Cho E.S., Maltby K.M., Wang Q., Jiang R. (2004). Odd-skipped related 2 (Osr2) encodes a key intrinsic regulator of secondary palate growth and morphogenesis. Development.

[35] Zhou J., Gao Y., Lan Y., Jia S., Jiang R. (2013). Pax9 regulates a molecular network involving Bmp4, Fgf10, Shh signaling and the Osr2 transcription factor to control palate morphogenesis. Development.

[36] Han J., Mayo J., Xu X., Li J., Bringas P., Maas R.L., Rubenstein J.L., Chai Y. (2009). Indirect modulation of Shh signaling by Dlx5 affects the oral-nasal patterning of palate and rescues cleft palate in Msx1-null mice. Development.

[37] Cesario J.M., Landin Malt A., Deacon L.J., Sandberg M., Vogt D., Tang Z., Zhao Y., Brown S., Rubenstein J.L., Jeong J. (2015). Lhx6 and Lhx8 promote palate development through negative regulation of a cell cycle inhibitor gene, p57Kip2. Hum. Mol. Genet..

[38] Iwata J., Suzuki A., Yokota T., Ho T.V., Pelikan R., Urata M., Sanchez-Lara P.A., Chai Y. (2014). TGFbeta regulates epithelial-mesenchymal interactions through WNT signaling activity to control muscle development in the soft palate. Development.

[39] Zhang Z., Song Y., Zhao X., Zhang X., Fermin C., Chen Y. (2002). Rescue of cleft palate in Msx1-deficient mice by transgenic Bmp4 reveals a network of BMP and Shh signaling in the regulation of mammalian palatogenesis. Development.

[40] Liu W., Sun X., Braut A., Mishina Y., Behringer R.R., Mina M., Martin J.F. (2005). Distinct functions for Bmp signaling in lip and palate fusion in mice. Development.

[41] Xiong W., He F., Morikawa Y., Yu X., Zhang Z., Lan Y., Jiang R., Cserjesi P., Chen Y. (2009). Hand2 is required in the epithelium for palatogenesis in mice. Dev. Biol..

[42] Andl T., Ahn K., Kairo A., Chu E.Y., Wine-Lee L., Reddy S.T., Croft N.J., Cebra-Thomas J.A., Metzger D., Chambon P. (2004). Epithelial Bmpr1a regulates differentiation and proliferation in postnatal hair follicles and is essential for tooth development. Development.

[43] Li L., Lin M., Wang Y., Cserjesi P., Chen Z., Chen Y. (2011). BmprIa is required in mesenchymal tissue and has limited redundant function with BmprIb in tooth and palate development. Dev. Biol..

[44] Baek J.A., Lan Y., Liu H., Maltby K.M., Mishina Y., Jiang R. (2011). Bmpr1a signaling plays critical roles in palatal shelf growth and palatal bone formation. Dev. Biol..

[45] He F., Xiong W., Wang Y., Matsui M., Yu X., Chai Y., Klingensmith J., Chen Y. (2010). Modulation of BMP signaling by Noggin is required for the maintenance of palatal epithelial integrity during palatogenesis. Dev. Biol..

[46] Li C., Lan Y., Krumlauf R., Jiang R. (2017). Modulating Wnt Signaling Rescues Palate Morphogenesis in Pax9 Mutant Mice. J. Dent. Res..

[47] Jia S., Zhou J., Wee Y., Mikkola M.L., Schneider P., D’Souza R.N. (2017). Anti-EDAR Agonist Antibody Therapy Resolves Palate Defects in Pax9^−/−^ Mice. J. Dent. Res..

[48] Jia S., Zhou J., Fanelli C., Wee Y., Bonds J., Schneider P., Mues G., D’Souza R.N. (2017). Small-molecule Wnt agonists correct cleft palates in Pax9 mutant mice in utero. Development.

[49] Headon D.J., Overbeek P.A. (1999). Involvement of a novel Tnf receptor homologue in hair follicle induction. Nat. Genet..

[50] Hilliard S.A., Yu L., Gu S., Zhang Z., Chen Y.P. (2005). Regional regulation of palatal growth and patterning along the anterior-posterior axis in mice. J. Anat..

[51] Li Q., Ding J. (2007). Gene expression analysis reveals that formation of the mouse anterior secondary palate involves recruitment of cells from the posterior side. Int. J. Dev. Biol..

[52] Welsh I.C., O’Brien T.P. (2009). Signaling integration in the rugae growth zone directs sequential SHH signaling center formation during the rostral outgrowth of the palate. Dev. Biol..

[53] Pantalacci S., Prochazka J., Martin A., Rothova M., Lambert A., Bernard L., Charles C., Viriot L., Peterkova R., Laudet V. (2008). Patterning of palatal rugae through sequential addition reveals an anterior/posterior boundary in palatal development. BMC Dev. Biol..

[54] Yu L., Gu S., Alappat S., Song Y., Yan M., Zhang X., Zhang G., Jiang Y., Zhang Z., Zhang Y. (2005). Shox2-deficient mice exhibit a rare type of incomplete clefting of the secondary palate. Development.

[55] Liu W., Lan Y., Pauws E., Meester-Smoor M.A., Stanier P., Zwarthoff E.C., Jiang R. (2008). The Mn1 transcription factor acts upstream of Tbx22 and preferentially regulates posterior palate growth in mice. Development.

[56] Pauws E., Moore G.E., Stanier P. (2009). A functional haplotype variant in the TBX22 promoter is associated with cleft palate and ankyloglossia. J. Med. Genet..

[57] Liu Y., Wang M., Zhao W., Yuan X., Yang X., Li Y., Qiu M., Zhu X.J., Zhang Z. (2015). Gpr177-mediated Wnt Signaling Is Required for Secondary Palate Development. J. Dent. Res..

[58] He F., Xiong W., Yu X., Espinoza-Lewis R., Liu C., Gu S., Nishita M., Suzuki K., Yamada G., Minami Y. (2008). Wnt5a regulates directional cell migration and cell proliferation via Ror2-mediated noncanonical pathway in mammalian palate development. Development.

[59] Nishihara H., Kobayashi N., Kimura-Yoshida C., Yan K., Bormuth O., Ding Q., Nakanishi A., Sasaki T., Hirakawa M., Sumiyama K. (2016). Coordinately Co-opted Multiple Transposable Elements Constitute an Enhancer for wnt5a Expression in the Mammalian Secondary Palate. PLoS Genet..

[60] Almaidhan A., Cesario J., Landin Malt A., Zhao Y., Sharma N., Choi V., Jeong J. (2014). Neural crest-specific deletion of Ldb1 leads to cleft secondary palate with impaired palatal shelf elevation. BMC Dev. Biol..

[61] Gao Y., Lan Y., Ovitt C.E., Jiang R. (2009). Functional equivalence of the zinc finger transcription factors Osr1 and Osr2 in mouse development. Dev. Biol..

[62] Fu X., Xu J., Chaturvedi P., Liu H., Jiang R., Lan Y. (2017). Identification of Osr2 Transcriptional Target Genes in Palate Development. J. Dent. Res..

[63] Guo L., Degenstein L., Fuchs E. (1996). Keratinocyte growth factor is required for hair development but not for wound healing. Genes Dev..

[64] Ferguson M.W. (1988). Palate development. Development.

[65] Alappat S.R., Zhang Z., Suzuki K., Zhang X., Liu H., Jiang R., Yamada G., Chen Y. (2005). The cellular and molecular etiology of the cleft secondary palate in Fgf10 mutant mice. Dev. Biol..

[66] Casey L.M., Lan Y., Cho E.S., Maltby K.M., Gridley T., Jiang R. (2006). Jag2-Notch1 signaling regulates oral epithelial differentiation and palate development. Dev. Dyn..

[67] Ingraham C.R., Kinoshita A., Kondo S., Yang B., Sajan S., Trout K.J., Malik M.I., Dunnwald M., Goudy S.L., Lovett M. (2006). Abnormal skin, limb and craniofacial morphogenesis in mice deficient for interferon regulatory factor 6 (Irf6). Nat. Genet..

[68] Peyrard-Janvid M., Leslie E.J., Kousa Y.A., Smith T.L., Dunnwald M., Magnusson M., Lentz B.A., Unneberg P., Fransson I., Koillinen H.K. (2014). Dominant mutations in GRHL3 cause Van der Woude Syndrome and disrupt oral periderm development. Am. J. Hum. Genet..

[69] Richardson R.J., Dixon J., Malhotra S., Hardman M.J., Knowles L., Boot-Handford R.P., Shore P., Whitmarsh A., Dixon M.J. (2006). Irf6 is a key determinant of the keratinocyte proliferation-differentiation switch. Nat. Genet..

[70] Jiang R., Lan Y., Chapman H.D., Shawber C., Norton C.R., Serreze D.V., Weinmaster G., Gridley T. (1998). Defects in limb, craniofacial, and thymic development in Jagged2 mutant mice. Genes Dev..

[71] Fitchett J.E., Hay E.D. (1989). Medial edge epithelium transforms to mesenchyme after embryonic palatal shelves fuse. Dev. Biol..

[72] Richardson R.J., Dixon J., Jiang R., Dixon M.J. (2009). Integration of IRF6 and Jagged2 signalling is essential for controlling palatal adhesion and fusion competence. Hum. Mol. Genet..

[73] Mills A.A., Zheng B., Wang X.J., Vogel H., Roop D.R., Bradley A. (1999). p63 is a p53 homologue required for limb and epidermal morphogenesis. Nature.

[74] Yang A., Schweitzer R., Sun D., Kaghad M., Walker N., Bronson R.T., Tabin C., Sharpe A., Caput D., Crum C. (1999). p63 is essential for regenerative proliferation in limb, craniofacial and epithelial development. Nature.

[75] Thomason H.A., Zhou H., Kouwenhoven E.N., Dotto G.P., Restivo G., Nguyen B.C., Little H., Dixon M.J., van Bokhoven H., Dixon J. (2010). Cooperation between the transcription factors p63 and IRF6 is essential to prevent cleft palate in mice. J. Clin. Investig..

[76] Candi E., Rufini A., Terrinoni A., Giamboi-Miraglia A., Lena A.M., Mantovani R., Knight R., Melino G. (2007). DeltaNp63 regulates thymic development through enhanced expression of FgfR2 and Jag2. Proc. Natl. Acad. Sci. USA.

[77] Sasaki Y., Ishida S., Morimoto I., Yamashita T., Kojima T., Kihara C., Tanaka T., Imai K., Nakamura Y., Tokino T. (2002). The p53 family member genes are involved in the Notch signal pathway. J. Biol. Chem..

[78] Richardson R.J., Hammond N.L., Coulombe P.A., Saloranta C., Nousiainen H.O., Salonen R., Berry A., Hanley N., Headon D., Karikoski R. (2014). Periderm prevents pathological epithelial adhesions during embryogenesis. J. Clin. Investig..

[79] Funato N., Nakamura M., Richardson J.A., Srivastava D., Yanagisawa H. (2012). Tbx1 regulates oral epithelial adhesion and palatal development. Hum. Mol. Genet..

[80] Vaziri Sani F., Hallberg K., Harfe B.D., McMahon A.P., Linde A., Gritli-Linde A. (2005). Fate-mapping of the epithelial seam during palatal fusion rules out epithelial-mesenchymal transformation. Dev. Biol..

[81] Xu X., Han J., Ito Y., Bringas P., Urata M.M., Chai Y. (2006). Cell autonomous requirement for Tgfbr2 in the disappearance of medial edge epithelium during palatal fusion. Dev. Biol..

[82] Jin J.Z., Ding J. (2006). Analysis of cell migration, transdifferentiation and apoptosis during mouse secondary palate fusion. Development.

[83] Cecconi F., Alvarez-Bolado G., Meyer B.I., Roth K.A., Gruss P. (1998). Apaf1 (CED-4 homolog) regulates programmed cell death in mammalian development. Cell.

[84] Cuervo R., Covarrubias L. (2004). Death is the major fate of medial edge epithelial cells and the cause of basal lamina degradation during palatogenesis. Development.

[85] Martinez-Alvarez C., Blanco M.J., Perez R., Rabadan M.A., Aparicio M., Resel E., Martinez T., Nieto M.A. (2004). Snail family members and cell survival in physiological and pathological cleft palates. Dev. Biol..

[86] Jin J.Z., Ding J. (2006). Analysis of Meox-2 mutant mice reveals a novel postfusion-based cleft palate. Dev. Dyn..

[87] Proetzel G., Pawlowski S.A., Wiles M.V., Yin M., Boivin G.P., Howles P.N., Ding J., Ferguson M.W., Doetschman T. (1995). Transforming growth factor-beta 3 is required for secondary palate fusion. Nat. Genet..

[88] Kaartinen V., Voncken J.W., Shuler C., Warburton D., Bu D., Heisterkamp N., Groffen J. (1995). Abnormal lung development and cleft palate in mice lacking TGF-beta 3 indicates defects of epithelial-mesenchymal interaction. Nat. Genet..

[89] Kaartinen V., Cui X.M., Heisterkamp N., Groffen J., Shuler C.F. (1997). Transforming growth factor-beta3 regulates transdifferentiation of medial edge epithelium during palatal fusion and associated degradation of the basement membrane. Dev. Dyn..

[90] Cui X.M., Shiomi N., Chen J., Saito T., Yamamoto T., Ito Y., Bringas P., Chai Y., Shuler C.F. (2005). Overexpression of Smad2 in Tgf-beta3-null mutant mice rescues cleft palate. Dev. Biol..

[91] Shiomi N., Cui X.M., Yamamoto T., Saito T., Shuler C.F. (2006). Inhibition of SMAD2 expression prevents murine palatal fusion. Dev. Dyn..

[92] Xu X., Han J., Ito Y., Bringas P., Deng C., Chai Y. (2008). Ectodermal Smad4 and p38 MAPK are functionally redundant in mediating TGF-beta/BMP signaling during tooth and palate development. Dev. Cell.

[93] Iwata J., Suzuki A., Pelikan R.C., Ho T.V., Sanchez-Lara P.A., Urata M., Dixon M.J., Chai Y. (2013). Smad4-Irf6 genetic interaction and TGFbeta-mediated IRF6 signaling cascade are crucial for palatal fusion in mice. Development.

[94] Shin J.O., Lee J.M., Bok J., Jung H.S. (2018). Inhibition of the Zeb family prevents murine palatogenesis through regulation of apoptosis and the cell cycle. Biochem. Biophys. Res. Commun..

[95] Jin J.Z., Warner D.R., Lu Q., Pisano M.M., Greene R.M., Ding J. (2014). Deciphering TGF-beta3 function in medial edge epithelium specification and fusion during mouse secondary palate development. Dev. Dyn..

[96] He F., Xiong W., Wang Y., Li L., Liu C., Yamagami T., Taketo M.M., Zhou C., Chen Y. (2011). Epithelial Wnt/beta-catenin signaling regulates palatal shelf fusion through regulation of Tgfbeta3 expression. Dev. Biol..

[97] Murray S.A., Oram K.F., Gridley T. (2007). Multiple functions of Snail family genes during palate development in mice. Development.

[98] Ke C.Y., Xiao W.L., Chen C.M., Lo L.J., Wong F.H. (2015). IRF6 is the mediator of TGFbeta3 during regulation of the epithelial mesenchymal transition and palatal fusion. Sci. Rep..

[99] Serrano M.J., Liu J., Svoboda K.K., Nawshad A., Benson M.D. (2015). Ephrin reverse signaling mediates palatal fusion and epithelial-to-mesenchymal transition independently of Tgfss3. J. Cell. Physiol..

[100] Mima J., Koshino A., Oka K., Uchida H., Hieda Y., Nohara K., Kogo M., Chai Y., Sakai T. (2013). Regulation of the epithelial adhesion molecule CEACAM1 is important for palate formation. PLoS ONE.

[101] Shin J.O., Nakagawa E., Kim E.J., Cho K.W., Lee J.M., Cho S.W., Jung H.S. (2012). miR-200b regulates cell migration via Zeb family during mouse palate development. Histochem. Cell Biol..

[102] Shin J.O., Lee J.M., Cho K.W., Kwak S., Kwon H.J., Lee M.J., Cho S.W., Kim K.S., Jung H.S. (2012). MiR-200b is involved in Tgf-beta signaling to regulate mammalian palate development. Histochem. Cell Biol..

[103] Park S.M., Gaur A.B., Lengyel E., Peter M.E. (2008). The miR-200 family determines the epithelial phenotype of cancer cells by targeting the E-cadherin repressors ZEB1 and ZEB2. Genes Dev..

[104] Li J., Rodriguez G., Han X., Janeckova E., Kahng S., Song B., Chai Y. (2019). Regulatory Mechanisms of Soft Palate Development and Malformations. J. Dent. Res..

[105] Li H., Jones K.L., Hooper J.E., Williams T. (2019). The molecular anatomy of mammalian upper lip and primary palate fusion at single cell resolution. Development.

